# Biological response modifier glucan through balancing of blood glucose may have a prophylactic potential in COVID-19 patients

**DOI:** 10.1007/s40200-020-00664-4

**Published:** 2020-10-21

**Authors:** Nobunao Ikewaki, Masaru Iwasaki, Samuel J. K. Abraham

**Affiliations:** 1 Department of Medical Life Science, Kyushu University of Health and Welfare, Nobeoka, Japan; 2 Institute of Immunology, Junsei Educational Institute, Nobeoka, Miyazaki Japan; 3 Yamanashi University- School of Medicine, Yamanashi Chuo, Japan; 4 Edogawa Evolutionary Laboratory of Science (EELS), Edogawa Hospital, Tokyo, Japan; 5 The Mary-Yoshio Translational Hexagon (MYTH), Nichi-In Centre for Regenerative Medicine (NCRM), Chennai, India; 6 GN Corporation Co. Ltd, 3-8, Wakamatsu, 400-0866 Kofu, Japan

**Keywords:** COVID-19, Biological response modifier glucan (BRMG), Fasting plasma glucose (FPG), Diabetes, Prophylaxis

## Abstract

With the COVID-19 pandemic causing huge threat to public health and definite treatment modalities and preventive vaccines yet to be arrived at, some of the key indicators of relevance to its prognosis have started emerging. One such independent predictor of outcome has been fasting plasma glucose (FPG) at the time of admission. Earlier, co-morbidities such as diabetes also have been reported to have a risk of relatively increased mortality due to COVID-19. In this background, we herein report on the beneficial effects of Biological response modifier glucan (BRMG) secreted by the black yeast *Aureobasidium pullulans* AFO-202 which has been proven to bring under control blood sugar levels in human subjects and also has potential in enhancing & regulating the immune parameters in relevance to COVID-19. We further recommend that this BRMG be tried in clinical studies of COVID-19 to provide a prophylactic effect for validation.

## Introduction

Fasting plasma glucose (FPG) level at admission has been indicated as a strong independent predictor of mortality in COVID-19 affected patients [1.2]. Wang et al. published their report of 605 COVID-19 patients in whom fasting plasma glucose (FPG) level ≥ 7.00 mmol/L at admission was a strong independent predictor of 28-day mortality [1] and recommended glycaemic testing & control in all COVID-19 patients even where they have no pre-existing diabetes. Zhang et al. also reported about 461 COVID-19 patients in whom fasting plasma glucose (FPG) level ≥ 6.23 mmol/L at admission was a strong independent predictor of poor outcomes. They further suggested that acute hyperglycaemia may cause organ damage by inducing endothelial dysfunction and thrombosis, which may be one of the key underlying mechanisms connecting the higher FPG with poor outcome of COVID-19 [2].

## Biological response modifier glucans (BRMG)

Beta-D-glucans are highly conserved structural components of cell walls in yeast, fungi, or seaweed which are naturally occurring physiologically active compounds termed as biological response modifier glucans (BRMGs) [3]. BRMGs from barley, oats, or wheat consist of linear β-(1,3;1,4)-D-glucans while those from the cell walls of yeast, fungi, and some bacteria consist of β -(1,3)- or β -(1,4)-glucan backbone with either (1,2)- or (1,6)-linked β -glucopyranosyl side branches. The structure of a beta glucan shapes its associated biological activities [3]. Both oat and fungal BRMGs have been shown to have biological response modifying effects but the BRMG content is more in yeast than in sources such as oats and hence yeast BRMGs are believed to be having more functional activity [4]. The effects of BRMGs on metabolic syndrome has been well documented in animal experiments and clinical trials [5]. BRMGs form highly viscous solutions in the human gut thereby leading to delayed gastric emptying, which leads to dietary glucose to be absorbed more gradually. The shape of the plasma glucose response curve is much flatter after BRMG intake. This in in turn contributes to the effects of lowering postprandial glucose and insulin responses, potentiating the feelings of satiety [5]. BRMGs also improve insulin sensitivity [6]. BRMGs have been shown to downregulate blood glucose by suppressing sodium-glucose transporter-1 expression in intestinal mucosa and by promoting glycogen synthesis along with inhibiting fat accumulation in the liver [7]. BRMGs help in maintaining glucose levels by forming a barrier in the small intestine, which prevents absorption of glucose and other nutrients, thereby reducing glycaemia, insulinaemia and cholesterol serum levels [8]. Decreased PI3K/Akt activity plays a key role in the pathogenesis of diabetes. BRMGs increase PI3K/Akt through several receptors, including Dectin-1, complement receptor 3, lactosylceramide and scavenger and toll-like receptors [5]. The effects on dyslipidaemia is attributed to the action of BRMG in altering bile acid excretion and the composition of bile acid pool. Increased bile acid excretion and increased activity of cholesterol 7 α -hydrolase, a major enzyme leads to cholesterol elimination in the body. BRMG can decrease the reabsorption of bile acids and increase their transport towards the large intestine [6] thus contributing to lessen the effects of metabolic diseases.

## AFO-202 derived BRMG & effect on FPG levels

Given this background on BRMGs, we wish to refer to a BRMG derived from the AFO-202 strain of *Aureobasidium pullulans.* This BRMG is secreted as an exo-polysacchride by the AFO-202 black yeast and hence does not involve additional purification or extraction processes, thus giving a highly pure BRMG [9]. The purity of a BRMG determines its functionality [10]. Dectin-1 having been identified as a receptor to regulate the PI3K/Akt activity leading to anti-diabetogenic effects of BRMG [11], it is noteworthy that, Dectin-1 is the key receptor for the AFO-202 BRMG to exert its biological actions [9].

We wish to highlight three clinical studies of this AFO-202 BRMG in relevance to its action of bringing down high FPG levels to normal values in patients with diabetes while maintaining the levels at a normal standard in healthy volunteers. The first study involved 22 healthy volunteers for whom regular consumption of this AFO-202 BRMG for three months helped to maintain FPG levels in subjects whose levels were within the normal standard while decreasing the high levels to normal values in 95% of the subjects who had high FPG at the start of the study [12]. In another study, when this AFO-202 BRMG was added to rice and consumed by 10 healthy male and female volunteers, significant inhibition of blood sugar elevation was observed at 60 minutes and 120 minutes after glucose load [12]. In a case study, three patients who consumed this AFO-202 BRMG for two months had baseline FPG levels of 171 mg/dl (9.4905 mmol/l), 114 mg/dl (6.327 mmol/l) and 129 mg/dl (7.1595 mmol/l), which decreased to 106 mg/dl (5.883 mmol/l), 103 mg/dl (5.7165 mmol/l) and 85 mg/dl (4.7175 mmol/l) at the end of the study. In the same three subjects, post-prandial blood glucose level decreased from 244 mg/dl (13.542 mmol/l) at baseline to 203 mg/dl (11.2665 mmol/l), 192 mg/dl (10.656 mmol/l) to 111 mg/dl (6.1605 mmol/l) and 252 mg/dl (13.986 mmol/l) to 118 mg/dl (6.549 mmol/l), respectively, at the end of the study [13]. The National Institute for Clinical Excellence (NICE) guidelines of the UK recommend FPG values of 4.0 to 5.9 mmol/L and 90 minutes after meal (post-prandial) values of under 7.8 mmol/L in non-diabetic individuals as normal [14].

## Mechanisms of AFO-202 BRMG’s anti-glycation effects in COVID-19

The possible role of AFO-202 BRMG’s anti-glycation effect results from its anti-inflammatory effect in diabetes. Patients with diabetes generally have a weakened immune system [15, 16], and BRMG can enhance the immune system [9]. The immunity-enhancing effect of BRMG is interconnected with its possible anti-glycation effect (normalization of HbA1c) in patients with diabetes. Beyond acting on blood glucose levels, this AFO-202 BRMG has been indicated as worthwhile to be considered for clinical trials in COVID-19 patients due to its balanced immune enhancement by decreasing hyper-inflammation factors such as IL-6 that lead to cytokine storms; increasing IFN-γ, sFAS, and factors like IL-7; and enhancing anti-viral cytotoxic immunity, as mediated by T cells, NK cells, macrophages, and antibody production by B cells [17]. Now, with Zhang et al. [2] having reported that high FPG level is a precipitating factor leading to poor COVID-19 outcomes, this BRMG has gained further significance as a prophylactic agent whose continuous supplementation has also been proven to balance FPG levels. In addition, they also play a vital role by both enhancing individuals’ immune capabilities to tackle COVID-19 infection and downregulating the factors responsible for hyperinflammation leading to cytokine storms, thereby preventing morbidity and mortality. Advanced glycation end-products (AGEs) that accumulate in diabetic and ageing tissues elicit a wide range of cell-mediated responses, leading to vascular dysfunction and atherosclerosis [15, 18]. With the renin-angiotensin (RAS) signalling pathway, oxidative stress and cell death, cytokine storm and endothelial dysfunction being implicated as the four major pathways involved in the pathogenesis of COVID-19, BRMGs have also been shown to help alleviate oxidative stress [19]. Diabetes is associated with persistent chronic inflammation of beta cells (β cells) in the pancreas caused by glycation [20] and with increased haemoglobin A1C (HbA1C), i.e., glycated haemoglobin levels with clinical significance as an indicator of diabetes diagnosis and glycaemic control. Three processes are presumed to underlie the pathogenesis of diabetes and glycation: persistent chronic inflammation, decreased function of vascular endothelial cells and decreased immune function [21]. Proper control of these factors can lead to the normalization of HbA1C levels and even improvement of diabetes. Further, increased blood sugar levels increased levels of clotting factors and relative inhibition of the fibrinolytic system, endothelial dysfunction, and enhanced platelet aggregation and activation, favouring a hypercoagulable pro-thrombotic state thus increasing the risk of coagulopathy due to COVID-19 [22]. Yeast BRMGs have been found to act on cytokine production regulating coagulation activation along with reversing diabetes-induced oxidative stress causing disturbances in the clotting parameters due to enhanced platelet aggregation and increased thrombin levels [23]. The diabetics are at heightened risk of disease severity in COVID-19 because of the higher rate of inflammatory processes seen in these individuals due to constant glucose recognition by C type lectin receptors [24]. Immunomodulatory effects of BRMGs resulting in increased resistance to both viral and bacterial infections have been attributed to their signalling mediated by CLR family of receptors [25]. Dectin-1 is a member of the CLR family [26] and we wish to reiterate that the AFO-202’s BRMG’s key receptor for exerting its biological activity is Dectin-1 [9]. It is interesting to specify that the beneficial effects of the AFO-202 BRMG as a prophylactic supplement to help combat coagulopathy associated with COVID-19, especially in vulnerable people like those with diabetes has been recently published [27].

## Conclusions

Especially at this time, when there is no definite treatment for COVID-19-infected patients or a preventative vaccine available, safe measures such as BRMGs that may provide a prophylactic effect should undergo clinical studies for further validation so that their consumption can help high-risk individuals to keep their FPG levels under control, thereby providing them with a safety net. Further research on the specific mechanisms of BRMGs on metabolic and immune system pathways are necessary to validate their beneficial effects in those with co-morbidities that make them prone to COVID-19 & complications.

## References

[1] Wang S, Ma P, Zhang S, et al. Fasting blood glucose at admission is an independent predictor for 28-day mortality in patients with COVID-19 without previous diagnosis of diabetes: a multi-centre retrospective study. Diabetologia. 2020;63(10):2102-2111. 10.1007/s00125-020-05209-1 PMC7347402 32647915

[2] Zhang B, Liu S, Zhang L, Dong Y, Zhang S. Admission fasting blood glucose predicts 30-day poor outcome in patients hospitalized for COVID-19 pneumonia. Diabetes Obes Metab. 2020. 10.1111/dom.14132. 10.1111/dom.14132 PMC7361510 32627338

[3] Novak M, Vetvicka V (2009). Glucans as biological response modifiers. Endocr Metab Immune Disord Drug Targets.

[4] Sobieralski K, Siwulski M, Lisiecka J, JĊdryczka M, Sas-Golak I, FruĪyĔska-JóĨwiak D (2012). Fungi-Derived β-Glucans as a component of functional food. Acta Sci Pol Hortorum Cultus.

[5] Chen J, Raymond K (2008). Beta-glucans in the treatment of diabetes and associated cardiovascular risks. Vasc Health Risk Manag.

[6] El Khoury D, Cuda C, Luhovyy BL, Anderson GH (2012). Beta glucan: health benefits in obesity and metabolic syndrome. J Nutr Metab.

[7] Cao Y, Zou S, Xu H (2016). Hypoglycemic activity of the Baker’s yeast β-glucan in obese/type 2 diabetic mice and the underlying mechanism. Mol Nutr Food Res.

[8] Francelino Andrade E, Vieira Lobato R, Vasques Araújo T (2014). Effect of beta-glucans in the control of blood glucose levels of diabetic patients: a systematic review. Nutr Hosp.

[9] Ikewaki N, Fujii N, Onaka T, Ikewaki S, Inoko H (2007). Immunological actions of Sophy beta-glucan (beta-1,3 – 1,6 glucan), currently available commercially as a health food supplement. Microbiol Immunol.

[10] Vetvicka V, Vetvickova J (2016). Comparison of immunological effects of commercially available beta-glucans: part III. Int Clin Pathol J.

[11] Li X, Wang J, Wang W, Liu C, Sun S, Gu J, Wang X, Boraschi D, Huang Y, Qu D (2013). Immunomodulatory activity of a novel, synthetic beta-glucan (β-glu6) in murine macrophages and human peripheral blood mononuclear cells. PLoS One.

[12] Yano et al. Sophy Beta-Glucan is effective in alleviating increased blood sugar levels. Abstract presented at the 55th Conference of the Japanese Society of Nutrition and Dietetics 2008.

[13] Dedeepiya VD, Sivaraman G, Venkatesh AP (2012). Potential effects of nichi glucan as a food supplement for diabetes mellitus and hyperlipidemia: preliminary findings from the study on three patients from India. Case Rep Med.

[14] Type 2 diabetes: prevention in people at high risk. NICE Public Health Guideline 38 – NICE. Published July 12, 2012. (Available from https://www.nice.org.uk/guidance/ph38) [Accessed 1st Oct 2020]

[15] Kolluru GK, Bir SC, Kevil CG (2012). Endothelial dysfunction and diabetes: effects on angiogenesis, vascular remodeling, and wound healing. Int J Vasc Med.

[16] Geerlings SE, Hoepelman AI (1999). Immune dysfunction in patients with diabetes mellitus (DM). FEMS Immunol Med Microbiol.

[17] Rao KS, Suryaprakash V, Senthilkumar R (2020). Role of Immune Dysregulation in Increased Mortality Among a Specific Subset of COVID-19 Patients and Immune-Enhancement Strategies for Combatting Through Nutritional Supplements. Front Immunol.

[18] Vlassara H (1996). Advanced glycation end-products and atherosclerosis. Ann Med.

[19] Murphy EJ, Masterson C, Rezoagli E (2020). β-Glucan extracts from the same edible shiitake mushroom Lentinus edodes produce differential in-vitro immunomodulatory and pulmonary cytoprotective effects - Implications for coronavirus disease (COVID-19) immunotherapies. Sci Total Environ.

[20] Cerf ME (2013). Beta cell dysfunction and insulin resistance. Front Endocrinol (Lausanne).

[21] Singh VP, Bali A, Singh N, Jaggi AS (2014). Advanced glycation end products and diabetic complications. Korean J Physiol Pharmacol.

[22] Hussain A, Bhowmik B, do Vale Moreira NC. COVID-19 and diabetes: Knowledge in progress. Diabetes Res Clin Pract. 2020;162:108142. 10.1016/j.diabres.2020.108142 PMC7144611 32278764

[23] El-Kashoury, Fattah SM, Ramadan L, El-Denshary ES. The Role of Yeast Beta Glucan on Blood Coagulation in Streptozotocin-Induced Diabetes and Irradiated Rats. 2016. Available from https://www.semanticscholar.org/paper/The-Role-of-Yeast-Beta-Glucan-on-Blood-Coagulation-El-Kashoury-Fatah/6f68a9067831eebb0015c2961882d1e91a89e84f. [Accessed 1st October, 2020].

[24] de Lucena TMC, da Silva Santos AF, de Lima BR, de Albuquerque Borborema ME, de Azevêdo Silva J (2020). Mechanism of inflammatory response in associated comorbidities in COVID-19. Diabetes Metab Syndr.

[25] Petit J, Bailey EC, Wheeler RT, de Oliveira CAF, Forlenza M, Wiegertjes GF (2019). Studies Into β-Glucan Recognition in Fish Suggests a Key Role for the C-Type Lectin Pathway. Front Immunol.

[26] Adachi Y, Ishii T, Ikeda Y, Hoshino A, Tamura H, Aketagawa J, Tanaka S, Ohno N (2004). Characterization of beta-glucan recognition site on C-type lectin, dectin 1. Infect Immun.

[27] Ikewaki N, Rao K, Archibold AD et al. Coagulopathy associated with COVID-19 – Perspectives & Preventive strategies using a biological response modifier Glucan. Thrombosis J 2020;18(27). 10.1186/s12959-020-00239-6 10.1186/s12959-020-00239-6 PMC7563912 33082714

